# Supplementary material for: Chromothripsis during telomere crisis is independent of NHEJ, and consistent with a replicative origin
Source: Genome Res. 2019 May;29(5):737–49. doi: 10.1101/gr.240705.118 (PMC6499312; doi:10.1101/gr.240705.118)
Supplement: Supplemental Material [file supp_gr.240705.118_Supplemental_file_1.zip › contigs/annotated_contigs/DB79/contig.2.DB79_length_603_mean_cov_8.17412935323.docx]

**DB79_length_603_mean_cov_8.17412935323**

```
CACAGAACTGGAACAAATGGTATATCATATTTTGTGTCAATATATATCTGATGGGATTTAAACATTTTATAATTTATATTTAGGTACAT
 >chr7:115691598-115691974 + E=1e-214
GTGTGTGCTTATGTATCTACACACATATATATGAATCTATATTAGCATAATCTAGATCTATTTCATGGAGGAGGAGGAAAGAGTAGTAT

CAATAATAGTAAAAATAGCTACAAACATTTATTGAATATTTATATCAGACACTGTGGTCAGTACTTTTCATGCACTATTGATTTAATTT

TCCTCAGAATTTTTGATAGGTACTATGTTATCATCTACTTTTTTCAGATAATAAAACTAAGGATTAGGTAAGTAACTTTTAGTGAGCCA

CAGGTTCTCAGGGCCTT|ATG|AGCCACCGTGCCCAGCAACATCTAAGTATTTAACAGAGTGTGACTACCTGACACACTCCTACTAAGA
                  >chr7:115692760-115692990 + E=1e-126
AATGTCATGGGGGCCGGGCGCAGTGGCTCACGCCTGTAATCCCAGCACTTTGGGAGGCCGAGGTGGGCGGATCACGAGGTCAGGAGATC

GAGACCATCCTGGCTAACACGGTGAAACCCCGTCTCTACTAAAAAAATACAAAAAAATTAGCTGGGCTTGG
```
